# State and county level legislative approaches to address racial/ethnic health inequities in Maryland (2012–2021)

**DOI:** 10.3389/fpubh.2025.1473971

**Published:** 2025-01-23

**Authors:** Makeda Walelo, Kellee White Whilby

**Affiliations:** Department of Health Policy and Management, University of Maryland College Park School of Public Health, College Park, MD, United States

**Keywords:** racial health inequities, structural racism, racial disparities, state policy, local level policy, legislative approaches

## Abstract

**Introduction:**

Public policies and legislative approaches are used to address racial health inequities. While most recent studies examine federal and state-level legislative activity, a paucity of analyses characterize policies enacted in a single state and across local jurisdictions. To address this gap, we identify racial health equity policies in the state of Maryland and describe key features and themes.

**Methods:**

A legal mapping study and content analysis was conducted. Maryland policies and legislative activity adopted at the state or county level (2012–2021) were identified by systematically searching Westlaw and state and county government legislative databases. Information for each policy was ascertained and analyzed to identify content domains.

**Results:**

We identified 22 state-level policies and 10 county-level policies and actions that explicitly addressed racial health inequities. Six domains were identified: healthcare and public health cultural competence; disease-specific care and outcomes; access to healthcare services; social determinants of health; collection action and research infrastructure; and structural racism. At the state- and county- level, most policies pertained to the healthcare and public health cultural competence domain. Of Maryland’s 24 counties, only 8 (33%) passed health equity policies and implemented equity-specific policy priorities.

**Conclusion:**

This study provides a snapshot of the Maryland policy landscape and suggests an increasing prioritization of equity policy at the state and county levels. While policies address issues ranging from cultural competence to structural racism, policy content differed by level of jurisdiction. Future efforts to critically evaluate the impact of specific policies on health inequities are needed.

## Introduction

Public policy and legislative actions (e.g., implementing regulations, creating special task forces or committees, mandating executive orders, and developing resolutions) can be powerful strategies to address racial and population health inequities (1, 2). Policies can be enacted at the federal, state, and local level to advance health equity (3, 4). Prior studies have conducted analyses examining federal and state policies and legislative approaches that impact racial/ethnic inequities in all 50 U.S. states (5–7). For example, Young et al. reviewed nationwide state legislation targeting racial/ethnic disparities in health from 1975 to 2001 and demonstrated significant variation across states (2). Other researchers have examined legislative approaches such as the adoption of resolutions. Mendez et al. documented over 100 areas adopting resolutions and declarations of “racism as a public health crisis” across U.S. jurisdictions (8). The significant heterogeneity in population health outcomes observed by states, may be attributable to increasing polarization of U.S. states’ economic, social, environmental, and health policy (e.g., Medicaid guidelines, health insurance regulations) context (7, 9). Yet, there are a dearth of studies characterizing a single state’s policy and legislative activity addressing racial/ethnic health inequities.

Policymakers in state legislatures and local governments – counties, cities, and municipalities – have been responsive to enacting legislation and implementing policies designed to influence racial equity. Scholars assert that state and local governments are uniquely positioned to advance racial equity for several reasons (3, 6). State and local governments have broad authority to act to improve population health and mitigate public health challenges. Notably, state and local governments can leverage the capacity of resources, systems, and structures that impact racial health equity (10). For example, both state and local governments have responsibility over processes, policies, services, and programs that reinforce criminal justice, economic, educational, environmental, and transportation determinants that directly create equitable communities or reify inequities. Further, policy development at the local level is more likely to be grounded in the historical, social, economic, and cultural needs and goals of the community as well as the lived experiences of residents (11). However, describing policies and legislative activities that address racial/ethnic health inequities within a single state and across local, specifically county-level jurisdictions, are limited and warrant further critical analysis.

The state of Maryland ranks high, in comparison to other U.S. states, when considering racial/ethnic diversity of the population, median household income, health system performance, and proportion of residents identifying as Democrat or democratic leaning (12–14). Yet, large racial/ethnic inequities in many leading causes of death (e.g., cardiovascular disease, diabetes, cancer) where Black and Brown populations are disproportionately burdened have been documented in Maryland over the past 15 years (15). Given the imperative to reduce racial/ethnic disparities, our understanding of the nature and scope of policies to directly address racial/ethnic inequities in Maryland is scant. The present study was undertaken to address these knowledge gaps by using a legal epidemiologic approach to map state- and county-level policies and policy tools that explicitly address racial health equity in the state of Maryland from 2012 to 2021. The objectives of this study were to assess the health equity policy landscape within the state of Maryland over a 10-year period. To achieve this objective, we used legal epidemiology methods and conducted a descriptive surveillance analysis of enacted racial health equity-focused policies at the state and county level. Secondly, we conducted a content analysis capturing key features of policies and policy-adjacent activities. Considering the role that state- and county-level policies can play in guiding and promoting equity, critical insights about their scope can inform the implementation of future equity focused policy priorities. More importantly, characterizing the landscape of racial equity policies and legislative actions at a more granular level can be leveraged so that future studies can be designed to evaluate the impact of specific policies on population health.

## Methods

Legal epidemiology methods, comprising the systematic identification, collection, and analysis of information about a policy issue (1, 16, 17), were used to examine enacted policies that explicitly address racial/ethnic health disparities in the state of Maryland from 2012 to 2021. This period was selected to capture policy and legislative activity after the passage of the Maryland Health Improvement and Disparities Reduction Act of 2012 which established Health Enterprise Zones (18). The purpose of the Health Enterprise Zones was to reduce racial/ethnic and geographic health disparities by improving access to care (e.g., recruiting primary care physicians to medically underserved areas) and providing services to improve health behaviors (e.g., recruiting and deploying community health workers to provide health education and screening) (19).

We also conducted a policy surveillance assessment of Maryland local jurisdictions (e.g., county and city) to systematically collect legal data from legislative websites during the 2012–2021 time period (20).

### Search strategy

Policies at the state level were systematically identified by searching the legal database Westlaw Next (Thompson Reuters) and the state legislative database on the Maryland General Assembly website (21). Westlaw Next is a widely-used legal database allowing for comprehensive research of federal and state legal content (i.e., statutes, case law, court orders, regulations, administrative decisions and guidance). Despite completeness of information for federal and state legal research, it is not well-suited for county or local legal policy research, given that it does not include this level of information. We additionally searched the National Conference of State Legislatures (NCSL) website and resources [Health Disparities Legislation Brief (22) and State Approaches to Reducing Health Disparities Report (23)], and publicly available search engines for additional state-level policies. A structured keyword search, informed by the NCSL State Approaches to Reducing Health Disparities Report (23) was conducted using the following search strings: “disparities”; “racial health disparities”; “health disparities”; “health equity”; “ethnic disparities”’ “racial and ethnic health disparities”; “ethnic health disparities”; “determinants of health”; “social determinants of health”; “social determinants”; “minority health”; “health”; “disparity/ies”; “inequality/ies”’ “inequity/ies”; “equity”; “racial”; “social”; “justice”; “minority”; “cultural competency”; “black”; “African-American”; “Hispanic”; “Latino.” Results were then filtered for relevance to health. Each of the aforementioned terms was searched independently in the databases. Where applicable, duplicate results were removed from analysis (7).

To our knowledge, a legal database comparable to Westlaw Next that captures local jurisdictions (e.g., county-level or city-level) does not exist and the best approach to identify county-level policies is to search the respective jurisdiction’s legislative website (20). We systematically searched 24 Maryland county-level entities (which included 23 counties and the independent municipality of Baltimore city) legislative databases and government websites and implemented similar search procedures as described above for the state, to the best extent possible given the different functionalities of each county’s legislative database. Due to the lack of legislation at the county level, we additionally searched for the existence of other policy-adjacent activities (e.g., creation of a new position or establishment of new funding, a resolution declaring racism a public health crisis) de-novo policy creation by county executive offices and county legislative boards that additionally reflect equity related government-led actions and levers. Toward this end, an additional Google search was conducted that included the county name, the Boolean operator “+,” and the phrases “health equity” (e.g., “Carroll County, MD + health equity”) to further identify county-level policy and policy-adjacent activities. Two study team members, who have been trained in legal epidemiologic surveillance methods, independently searched the legislative databases, government websites, and public search engines for the state and county.

### Data extraction

Our eligibility criteria included: (1) explicitly referenced race, ethnicity, racial disparities, health equity, minority communities, and social justice; (2) explicit intent to address health or health care disparities/inequities or improve the health or health care of minoritized groups; (3) passed or enacted into law between 2010 and 2021. This study’s primary emphasis is on the state of Maryland and county-level policies that explicitly and directly named and addressed racial/ethnic health disparities and inequities. Policies that indirectly tackle disparities (e.g., minimum wage) were deemed outside the scope of the study and were excluded. For policies meeting the eligibility criteria, we collected and read the full text of each identified policy and policy-adjacent activity. After an initial appraisal, an additional review was conducted to determine if any other policies or initiatives were referenced. Using the information generated from the state and county searches, we qualitatively developed a data extraction tool to document and catalog in a structured data table the following information: policy name and number, hyperlink to the full text of the policy, effective date, and data related to domains related to health equity for the content analysis described below. Similar to prior research (20), data extracted for the content analysis were selected based on guidance from a review of the existing literature.

### Content analysis

We conducted an analysis to characterize policies and policy activity quantitatively (number of policies, frequency per year, and percentages) and qualitatively (topical descriptions of policy themes and attributes) (24). Descriptive statistical analysis (e.g., counts and percentages) was computed for state- and county-level policies and categorized by date of implementation and thematic policy attributes. The content of each policy was examined using a combination of deductive and inductive approaches (25), using the NCSL report to guide theme development from the content of the policies. The content analysis was conducted in two rounds by independent reviewers. Policy data was subsequently assessed for trends by state- or county-level and by policy domain. Policies and policy-adjacent activities were coded and categorized into specific domains according to their content – if the language in the bill text or activity represented the domain. Topical coding was not mutually exclusive, and policies could belong to more than one category, as policies often addressed multiple topic areas (2). Each policy was extensively reviewed by at least one researcher to be coded and categorized. This research was deemed exempt from an institutional review board because the research did not involve human data or participants.

## Results

During the 2012 to 2021 study period, we identified 22 state-level policies and 10 county-level policy actions, that explicitly addressed racial/ethnic health inequities in Maryland. Detailed summary information about each state- and county-level policy can be found in the Supplementary materials.

Of the 22 state-level policies identified during the 2012–2021 study time frame, more than half were passed in 2020 and 2021 (Table 1). Only 1 policy addressed structural racism, 10 addressed interpersonal racism and 11 did not address any level of racism. Approximately one-third of state-level policies included a mandate to collect equity related data. Most of the county policies and policy-adjacent activities were passed after 2019. Of the identified policies and activities, three addressed structural racism and seven addressed interpersonal racism. Most of the county-level policies and activities either established a new center, office, program, or workgroup and passed equity-centered resolutions (e.g., declaration of racism as a public health crisis).

**Table 1:** Summary of characteristics of Maryland state- and county-level policies.

		State (*n* = 22)		County (*n* = 10)	
		*n*	%	*n*	%
Year introduced	2012	3	13.6	0	0
	2013	0	0	0	0
	2014	0	0	0	0
	2015	1	4.5	0	0
	2016	0	0	0	0
	2017	2	9.1	0	0
	2018	3	13.6	1	10
	2019	1	4.5	1	10
	2020	3	13.6	5	50
	2021	9	40.9	3	30
Level of racism addressed	Structural / institutional	1	4.5	3	30
	Interpersonal	10	45.5	7	70
	None	11	50.0	0	0
Budget appropriated or allocated		6	27.3	1	10
Mandates data collection related to equity		8	36.4	1	10
Establishes new center, office, or program		2	9.1	4	40
Establishes a workgroup or commission		6	27.3	4	40
Passage of equity-centered resolutions		0	0	4	40

Table 2 presents examples of state and county-level policies for each of the following domains: (1) healthcare and public health cultural competence; (2) disease-specific care and outcomes; (3) access to healthcare services; (4) social determinants of health; (5) collective action and research infrastructure; and (6) structural racism. Policies coded in the healthcare and public health cultural competence domain identified efforts to train and develop the capacity of health care and public health professional’s knowledge, attitudes, skills, and behaviors to operate and perform in a culturally competent manner. Disease-specific care and outcomes pertained to specific disease or health conditions to be addressed among racial/ethnic groups. Policies coded in the access to healthcare services domain identified issues of availability, accessibility, accommodation, affordability, and acceptability of healthcare services addressed in specific racial/ethnic groups. The social determinants of health domain identified education, economic, neighborhood and built environment factors as fundamental causes of health inequity and targets of policy action to be addressed among specific racial/ethnic groups. Policies characterized in the collective action and research infrastructure domain established task forces, committees, community programs, and research initiatives aimed at addressing health inequities. Policies coded under the structural racism domain were characterized by the creation of policies, decision-making and budgetary processes and structures that mitigated the effects of discrimination across social systems and prevented the unintentional perpetuation of discriminatory beliefs, values, or the inequitable distribution of resources. The full list of state- and county-level policies by content domain is included in the Supplementary Tables S1–S3.

**Table 2:** Examples of state- and county-level policies by content domain.

Content domains	State	County
Healthcare and public health cultural competence Identifies efforts to train and develop the capacity of health care and public health professional’s knowledge, attitudes, skills, and behaviors to operate and perform in a culturally competent manner.	Public Health – Implicit Bias Training and the Office of Minority Health and Health Disparities-SB5/HB28 [2021].	Charles County: Establishment of Chief Equity Officer position in County Executive Office [2020].
Disease-specific care and outcomes Identifies a specific disease or health condition to be addressed among racial/ethnic groups.	Health – Maternal Mortality Review Program – Recommendations and Reporting Requirement - SB 356/HB583 [2019].	None identified.
Access to healthcare services Identifies issues of availability, accessibility, accommodation, affordability, and acceptability of healthcare services to be addressed in specific racial/ethnic groups.	Maryland Health Improvement and Disparities Reduction Act of 2012 - SB234/HB439 [2012].	Prince George’s County: Resolution CR-66-2020: Declaration of Racism as a Crisis of Public Health, Public Safety and Economic Welfare [2020].
Social determinants of health Identifies education, economic, neighborhood and built environment factors as fundamental causes of health inequity and targets of policy action to be addressed among specific racial/ethnic groups.	The Shirley Nathan-Pulliam Health Equity Act of 2021 – SB52/HB78 [2021].	Frederick County: Office of Equity and Inclusion and Equity and Inclusion Commission. (County Code Article 17 [XVII]) [2021].
Collective action and research infrastructure Establishes task forces, committees, community programs, and research initiatives aimed at addressing health inequities.	Health Services Cost Review Commission - Community Benefits - Reporting - HB1169 [2020].	St. Mary’s County: Created Joint Resolution to Advance Equity, a collaboration of the Sheriff’s Office, SMC Public Schools, and Health Department [2020].
Structural racism Identifies policy, decision-making and budgetary processes and structures that seek to mitigate the effects of discrimination across social systems and prevent the unintentional perpetuation of discriminatory beliefs, values, or inequitable distribution of resources.	Maryland Behavioral Health and Public Safety Center of Excellence - Establishment - HB 1280 [2021].	Montgomery County: Racial Equity and Social Justice Act [2019].

**Figure 1:**
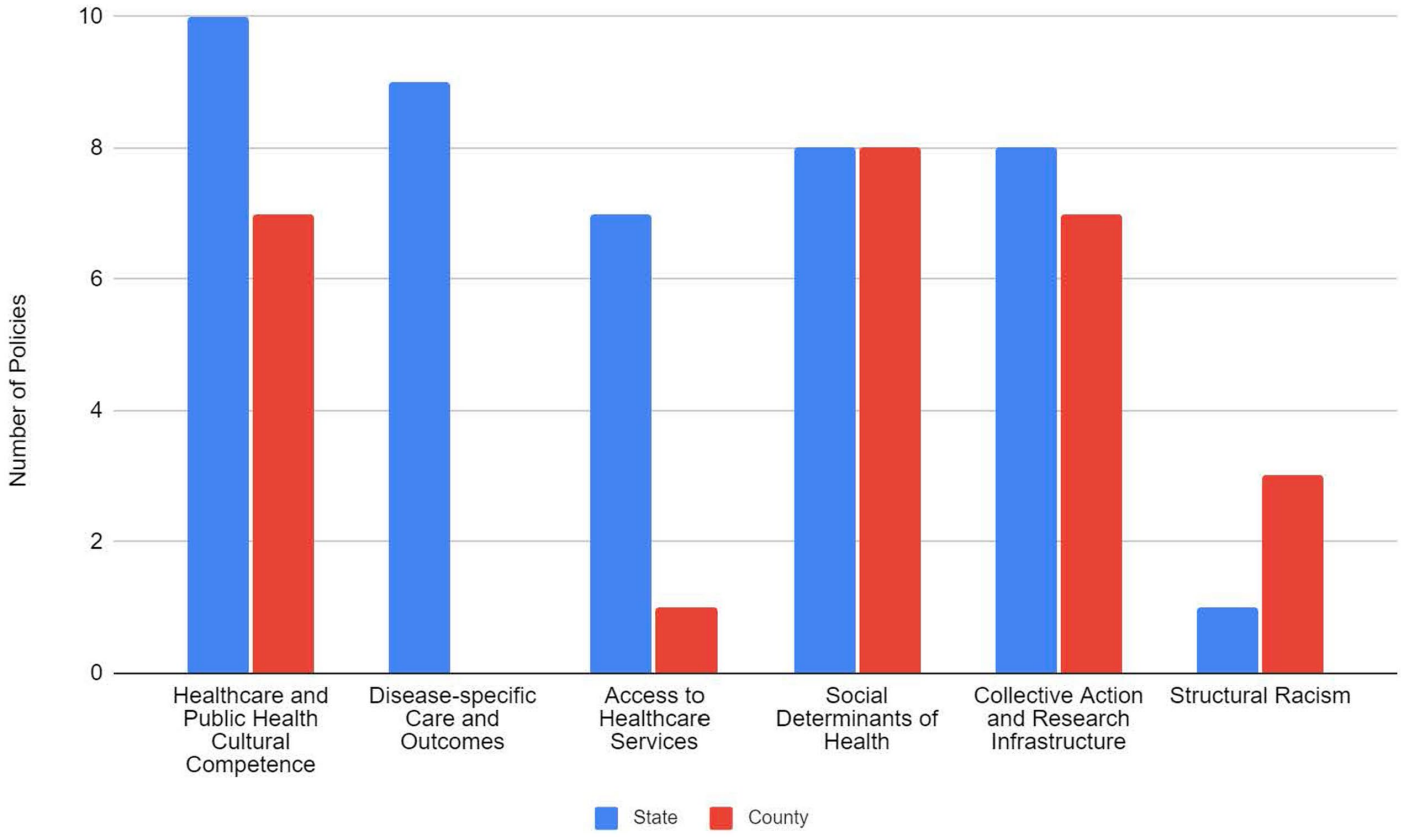
State and county-level health equity policy activity by content domain in Maryland, 2012 - 2021.

A summary of the number of policies at the state- and county-level by content domain is presented (Figure 1). At the state-level most policies were categorized in the healthcare and public health cultural competence and the disease-specific care and outcomes domains, with the fewest number of policies categorized in the structural racism domain. For counties, most of the policy activity was centered on three domains: social determinants of health; collective action and research infrastructure; and healthcare and public health cultural competence. Relatively fewer policies were categorized in the access to care and structural racism domains. There were no policy activities identified that addressed the disease-specific care and outcomes domain at the county-level.

**Figure 2:**
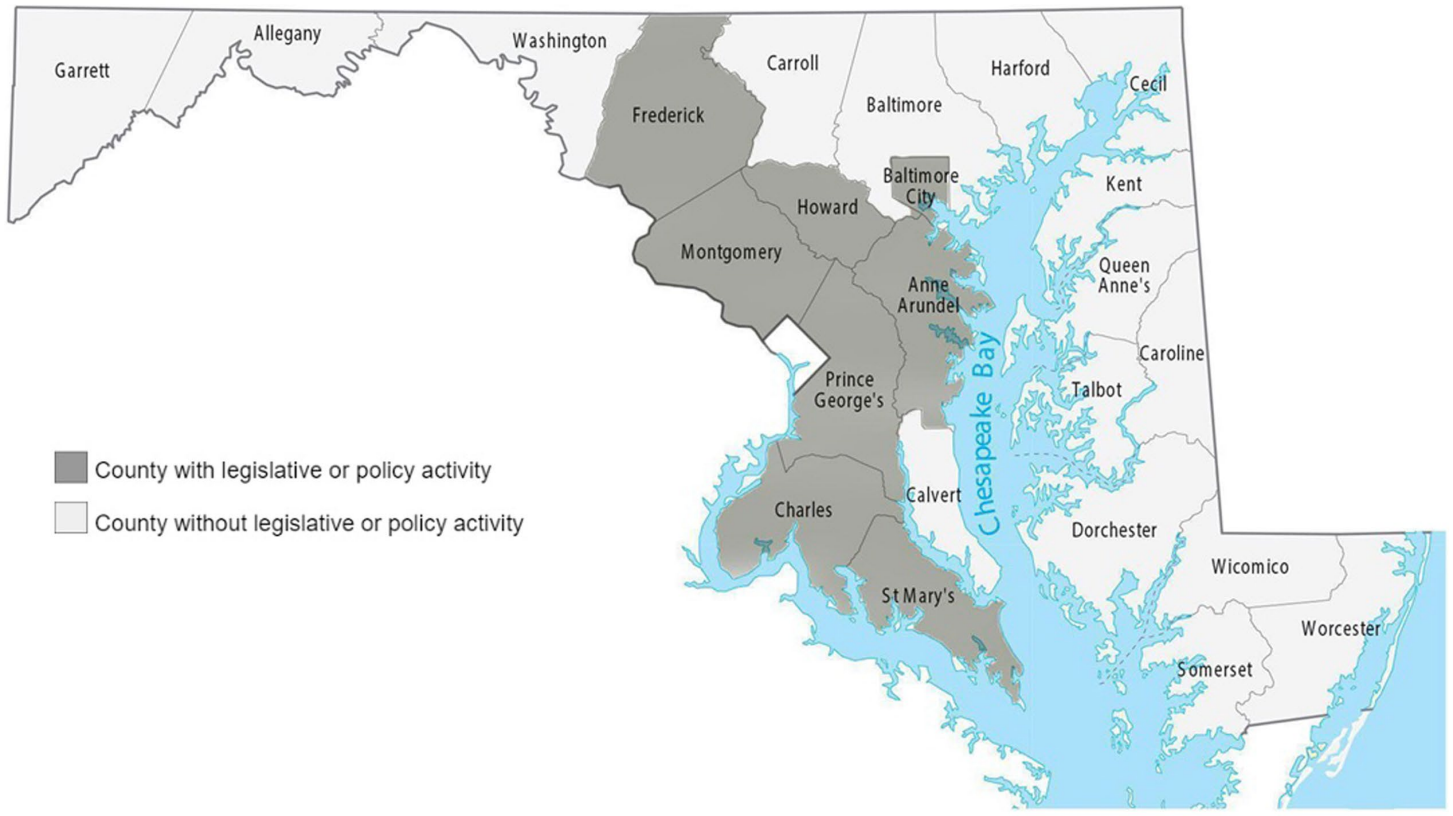
Map of Maryland counties by presence of legislative or policy activity.

Of the 24 counties in Maryland, only eight (33%) passed health equity policies and implemented equity-specific policy priorities. This policy activity was centralized among eight counties located in the central and southern regions of the state (i.e., Anne Arundel County, Baltimore City, Charles County, Frederick County, Howard County, Montgomery County, Prince George’s County, and St. Mary’s County), with no representation on the Eastern Shore or the western region of the state (Figure 2).

## Discussion

This study provides, to our knowledge, one of the first systematic assessments of the policies and policy-adjacent activity that explicitly seek to address racial/ethnic inequities in Maryland. We identified 22 state-level policies and 10 county-level policy and policy-adjacent activities, which provide a snapshot of the Maryland policy landscape and suggest an increasing prioritization of equity as a policy priority at both state and county levels. While policies tackle a breadth of equity issues ranging from cultural competency to structural racism, policy content differed by level of jurisdiction. Further, we observed significant heterogeneity in the scope of equity policies and activity passed at the county-level and found a lack of policies to address structural determinants of inequities at both the state- and county-level.

During the 12-year study period, we noted a relative increasing frequency of policies that specifically addressed racial health in the Maryland State House. In the Maryland General Assembly, more than half of all identified legislation was passed in the last 2 years (2020–2021) of the study period. This overlaps with a period of increasing social awareness and pressure from constituents, grassroots organizers, and community-based advocates to have decision-makers prioritize policies to address health disparities and solutions for equity. Also, this is consistent with a rise in policies in other states and at the federal level that have been proposed and enacted to address racial inequities (7). For example, a recent systematic policy review of black maternal health-related policies proposed federally and in Massachusetts found an increase in policies proposed and subsequently enacted to address racial disparities and health equity in maternal health between 2010 and 2020 (7).

We identified policies at the state-level in each of the 6 domains, with the majority of policies largely centered on improving healthcare and public health cultural competence as well as ameliorating disparities in disease-specific outcomes and health conditions. Action across these domains reflects the variety and interplay of the multitude of approaches necessary to create and foster healthy and equitable outcomes and communities. However, the observation that relatively fewer policies focused on addressing fundamental, structural barriers that are considered the root causes of racial inequities is noteworthy, particularly for advocates and policy and decision-makers. Greater consideration needs to be given to developing and implementing a policy agenda that prioritizes and remedies structural drivers of health inequity.

Our findings demonstrate substantial variation in the extent to which county-level governments are developing, enacting, and implementing policies and other legislative approaches that can move us to achieve health equity and reduce racial health inequities. Most of the policy activity was concentrated in counties located in the central and southern regions of the state with no activity in the western or eastern shore of the state. These results raise critical questions about the capacity and resources of county-level jurisdictions to develop and implement equity-focused policies. Many counties may have to balance the challenge of directly addressing health disparities with fiscal constraints and competing budgetary priorities. The variation in policy action at the county level may also be a function of intention to prioritize and be responsive to equity issues, particularly given the political polarization of policy context across Maryland jurisdictions. Partisan differences in the recognition and acknowledgment of racial/ethnic health inequities have been shown (26, 27). Political affiliation can influence the implementation and the scope of legislation enacted to make consequential changes toward addressing inequities. Further, there is a misalignment between county-level policy action versus county-level factors that shape the conditions where people live and contribute to better health. Eastern shore (e.g., Dorchester and Somerset) and northwestern counties (e.g., Allegany and Washington) where we did not observe any policy action have some of the lowest county health rankings on health behaviors, quality of life, clinical care, social and economic factors, in comparison to other counties that exhibited policy action (28). In particular, these are rural counties with greater proportions of poor black and poor white residents compared to areas where policy action occurred. Future research should seek to systematically understand county-level factors that influence the capacity to develop and implement racial equity policy.

The county-level had the most policy activity categorized in the structural racism domain. For example, resolutions from Montgomery, Frederick, and Prince George’s Counties, all identify specific objectives to incorporate antiracist principles into their governing practices; however, as resolutions, these policy actions function more as statements of support or intent than enforceable law. Most notable, in Montgomery County, the Racial Equity and Social Justice Act of 2019 mandated executive leadership (i.e., creation of chief equity officer), legislative committees on racial equity, and required the conduct of racial equity impact assessments for all legislative and budgetary priorities introduced in the county council. This represents a key example of the capacity of local governments to leverage resources and processes to drive structural changes related to facilitating racial equity in decision-making and resource allocation, and catalyze relationship across sectors and communities, which can ultimately lead to long-term systemic change (3, 10). However, it is often difficult for county governments to cull resources for data infrastructure as a standard of practice of surveillance to inform equity-centered public health praxis and foster accountability among decision-makers (29).

While we identified several equity centered policies at the county level, we additionally identified substantial policy-adjacent activity, which was not the result of legislation. Given that constituents are often told to vote for change, such that our elected officials approve policies that yield a positive impact, this finding highlights the relationship between public health and executive power. Most county-level health equity initiatives occurred due to executive action. As such, we may ask: what is the role of county executives in advancing health equity? With more than half of county-level initiatives that did not occur through the deliberative, legislative process– what does that mean for advancing health equity if so much change is affected by just a few individuals? This finding implies that there is a role for community and advocacy groups to influence the policy agenda of a given incumbent and that there may be space for increased collaboration via cross-sector partnerships (Change Lab Solutions Strategies for Equitable Policymaking). Overall, we find that at the sub-state level, there may be an important role for nonprofit and community-based actors to advance health equity in collaboration with county-level executive offices (1).

This study is, to our knowledge, one of the first to employ a systematic, approach to identify and analyze policy activity explicitly designed to advance health equity over 10 years in Maryland. We examined county-level policy activity to evaluate within-state heterogeneity and give a more detailed snapshot of the policy trends at the sub-state level, where much public health rulemaking authority is held. Given these strengths, it is important to consider a few limitations that may impact the overall interpretation of results. First, we did not have access to a comprehensive legal database comparable to Westlaw Next or LexisNexis StateNet for the analysis of policies. To our knowledge, such a database that can provide structured legal data at the local level for county-level policies in Maryland does not exist. While we used a variety of resources to get the most complete local policy information possible, we may have missed some county-level policies and initiatives due to the fallibility of online sources (e.g., lags in website updates). Second, this study derived data using legal epidemiological methods and only included laws passed by state legislatures. Therefore, it is possible that any jurisdiction in our analysis that addressed racial health equity through administrative regulations was not captured. Third, this study was limited to policies that were passed and did not include bills that were proposed and failed, vetoed, or un-funded policy items. Inclusion of these may offer some insight regarding challenges and latent trends experienced within the state that could be helpful in the future in implementing policy. Fourth, the criteria for inclusion did not include linguistic differences, and more importantly we did not include studies that indirectly influence health equity. Lastly, the selected themes in the content analysis may miss elements of the policies that address racial and health equity. While our approaches are robust enough for this nascent study, future studies should examine and compare policy activity in and across other states.

This study has implications for policy and practice because it provides foundational evidence to understand the scope and nature of Maryland state and county government policies that address racial health equity. This data can be used by policymakers and advocates at both the state and county levels to obtain information to improve the development and implementation of policies that influence disease distribution and mitigate racial/ethnic health inequities. Examining the extent to which the identified policies impacted population health and racial disparities was beyond the scope of this study. Rigorous evidence of effective policy action is limited and future directions of this work should estimate the efficacy of these policy efforts on the magnitude of health inequities. Also, it will be important to establish and understand the barriers and enablers of more equity-oriented government policy action at the state and county level.

In the past decade, there has been a growing proliferation of legislative activity, policies, and policy-adjacent activities to address racial health equity at the state and county level. Our objective was to capture and describe the spectrum of policy activity related to racial health equity. We find that in Maryland, policies and legislative activity to address racial/ethnic inequalities are diverse in the content domains covered. The majority of policy priorities focus on individual-level factors, followed by health system-related factors, with limited legislation focused on structural racism. Although these policy efforts are critical to address racial health equity, there remain several gaps to be addressed at the state- and county-level to improve health outcomes for Black, Brown, and indigenous populations. Policies that focus solely on individual-level factors may have limited efficacy in tangibly reducing and eliminating health inequities. It may be helpful for Maryland policymakers and advocates to build support for and prioritize a policy agenda that emphasizes structural factors that impact population health and further advance health equity. Efforts to promote strategic messaging and mobilization for racial equity policy across systems that shape economic, educational, employment, and housing opportunities have the potential to accelerate and maximize public and policymaker support for policies that foster greater equity (30). With increasing awareness in how state legislative action impacts health policy, there is an opportunity at both state and county levels to implement innovative policy solutions and employ policy tools to eliminate racial/ethnic health inequities.

## Data Availability

The original contributions presented in the study are included in the article/Supplementary material, further inquiries can be directed to the corresponding author.
