# Supplementary material for: State and county level legislative approaches to address racial/ethnic health inequities in Maryland (2012–2021)
Source: Front Public Health. 2025 Jan 23;13:1473971. doi: 10.3389/fpubh.2025.1473971 (PMC11798927; doi:10.3389/fpubh.2025.1473971)
Supplement: Supplementary file 1 [file Table_1.docx]

**Supplementary Materials**

**Table S.1:** County-Level Health Equity Activity by Content Domain

| **Healthcare and Public Health Cultural Competence** | **Disease-specific Care and Outcomes** | **Social Determinants of Health** | **Collective Action and Research Infrastructure** | **Structural Racism** |
| --- | --- | --- | --- | --- |
| Baltimore City (2018): Equity Assessment Ordinance (Baltimore City Code, Article 1 39-1) | Prince George’s County (2020): Resolution to Declare Racism a Crisis of Public Health (Resolution CR-66-2020) | Anne Arundel County (2020): Established the Office of Health Equity and Racial Justice | Anne Arundel County (2020): Established the Office of Health Equity and Racial Justice | Frederick County (2020): Joint Declaration of Racism as Public Health Crisis |
| Charles County (2020): Establishment of Chief Equity Officer position in County Executive Office |  | Baltimore City (2018): Equity Assessment Ordinance (Baltimore City Code, Article 1 39-1) | Charles County (2020): Establishment of Chief Equity Officer position in County Executive Office | Montgomery County (2019): Racial Equity and Social Justice Act |
| Howard County (2021): Establishment of LGBTQIA+ Commission and La Alianza Latina Commission in County Council (County Code Sec. 12.218 - 12.219) |  | Frederick County (2021): Office of Equity and Inclusion and Equity and Inclusion Commission. (County Code Article 17 [XVII]) | Frederick County (2021): Office of Equity and Inclusion and Equity and Inclusion Commission. (County Code Article 17 [XVII]) | Montgomery County (2020): Resolution: Declaration of Racism as a Public Health Crisis |
| St. Mary’s County (2020): Created Joint Resolution to Advance Equity, a collaboration of the Sheriff’s Office, SMC Public Schools, and Health Department. |  | Howard County (2021): Establishment of LGBTQIA+ Commission and La Alianza Latina Commission in County Council (County Code Sec. 12.218 - 12.219) | Howard County (2021): Establishment of LGBTQIA+ Commission and La Alianza Latina Commission in County Council (County Code Sec. 12.218 - 12.219) | Prince George’s County (2020): Resolution to Declare Racism a Crisis of Public Health (Resolution CR-66-2020) |
|  |  | Montgomery County (2019): Racial Equity and Social Justice Act | Montgomery County (2019): Racial Equity and Social Justice Act |  |
|  |  | St. Mary’s County (2020): Created Joint Resolution to Advance Equity, a collaboration of the Sheriff’s Office, SMC Public Schools, and Health Department. | St. Mary’s County (2020): Created Joint Resolution to Advance Equity, a collaboration of the Sheriff’s Office, SMC Public Schools, and Health Department. |  |

**Table S.2:** State-Level Health Equity Legislative Activity

| **Task Force or Workgroup Created** | **Budget Allocation Required** | **Center or Permanent Program Created** | **New Regulations Established** |
| --- | --- | --- | --- |
| 2012: Maryland Health Improvement and Disparities Reduction Act of 2012 - SB234/HB439 | 2012: Maryland Health Improvement and Disparities Reduction Act of 2012 - SB234/HB439 | 2021: Maryland Behavioral Health and Public Safety Center of Excellence - Establishment - HB 1280 | 2012: Cultural Competency and Health Literacy – Education |
| 2017: University of Maryland School of Public Health, Center for Health Equity – Workgroup on Health in All Policies - HB1225/SB340 | 2018: Maryland Health Care Commission – Mortality Rates of African American Infants and Infants in Rural Areas – Study - HB 716/SB 266 | 2021: Maryland Health Equity Resource Act - SB172/HB463 | 2012: Maryland Health Improvement and Disparities Reduction Act of 2012 - SB234/HB439 |
| 2018: Task Force on the Social Determinants of Health in Baltimore City - SB444 | 2021: Maryland Health Equity Resource Act - SB172/HB463 |  | 2015: Health Care Disparities, Cultural and Linguistic Competency, and Health Literacy – Continuing Education - HB580/SB198 |
| 2020: Health Services Cost Review Commission - Community Benefits - Reporting - HB1169 | 2021: Public Health – Implicit Bias Training and the Office of Minority Health and Health Disparities-SB5/HB28 |  | 2017: University of Maryland School of Public Health, Center for Health Equity – Workgroup on Health in All Policies - HB1225/SB340 |
| 2021: The Shirley Nathan–Pulliam Health Equity Act of 2021 -SB 52/HB78 | 2021: The Shirley Nathan–Pulliam Health Equity Act of 2021 -SB 52/HB78 |  | 2017: Health Occupations Boards – Racial and Ethnic Health Disparities – Information Campaigns – Reporting - SB503 |
| 2021: Maryland Food System Resiliency Council - HB831 | 2021: Recovery for the Economy, Livelihoods, Industries, Entrepreneurs, and Families (RELIEF) Act -SB 496 |  | 2018: Public Health - Maternal Mortality Review Program - Report and Stakeholder Meetings - HB1518 |
|  |  |  | 2019: Health – Maternal Mortality Review Program – Recommendations and Reporting Requirement - SB 356/HB583 |
|  |  |  | 2021: Maryland Office of Minority Health and Health Disparities and Maryland Health Care Commission – Reporting Requirements - SB796 |
|  |  |  | 2021: Public Health – Data – Race and Ethnicity Information - SB 565/HB309 |
|  |  |  | 2021: Maryland Health Equity Resource Act - SB172/HB463 |
|  |  |  | 2021: Public Health – Implicit Bias Training and the Office of Minority Health and Health Disparities-SB5/HB28 |

**Table S.3:** State-Level Health Equity Policies by Content Domain

| **Healthcare and Public Health Cultural Competence** | **Disease-specific Care and Outcomes** | **Access to Healthcare Services** | **Social Determinants of Health** | **Collective Action and Research Infrastructure** | **Structural Racism** |
| --- | --- | --- | --- | --- | --- |
| 2012: Cultural Competency and Health Literacy – Education | 2012: Hepatitis B and Hepatitis C Viruses – Public Awareness, Treatment, and Outreach - HB 641 | 2012: Maryland Health Improvement and Disparities Reduction Act of 2012 - SB234/HB439 | 2012: Maryland Health Improvement and Disparities Reduction Act of 2012 - SB234/HB439 | 2012: Hepatitis B and Hepatitis C Viruses – Public Awareness, Treatment, and Outreach - HB 641 | 2021: Maryland Behavioral Health and Public Safety Center of Excellence - Establishment - HB 1280 |
| 2015: Health Care Disparities, Cultural and Linguistic Competency, and Health Literacy – Continuing Education - HB580/SB198 | 2018: Public Health - Maternal Mortality Review Program - Report and Stakeholder Meetings - HB1518 | 2012: Hepatitis B and Hepatitis C Viruses – Public Awareness, Treatment, and Outreach - HB 641 | 2018: Task Force on the Social Determinants of Health in Baltimore City - SB444 | 2012: Maryland Health Improvement and Disparities Reduction Act of 2012 - SB234/HB439 |  |
| 2017: Health Occupations Boards – Racial and Ethnic Health Disparities – Information Campaigns – Reporting - SB503 | 2018: Maryland Health Care Commission – Mortality Rates of African American Infants and Infants in Rural Areas – Study - HB 716/SB 266 | 2020: Health Services Cost Review Commission - Community Benefits - Reporting - HB1169 | 2019: Health – Maternal Mortality Review Program – Recommendations and Reporting Requirement - SB 356/HB583 | 2015: Health Care Disparities, Cultural and Linguistic Competency, and Health Literacy – Continuing Education - HB580/SB198 |  |
| 2021: Maryland Office of Minority Health and Health Disparities and Maryland Health Care Commission – Reporting Requirements - SB796 | 2019: Health – Maternal Mortality Review Program – Recommendations and Reporting Requirement - SB 356/HB583 | 2021: Maryland Health Equity Resource Act - SB172/HB463 | 2020: Health Services Cost Review Commission - Community Benefits - Reporting - HB1169 | 2017: University of Maryland School of Public Health, Center for Health Equity – Workgroup on Health in All Policies - HB1225/SB340 |  |
| 2021: Public Health – Data – Race and Ethnicity Information - SB 565/HB309 | 2020: Public Health - Maternal Mortality and Morbidity - Implicit Bias Training and Study - HB837 | 2021: Preserve Telehealth Access Act of 2021 - HB 123/SB3 | 2021: The Shirley Nathan–Pulliam Health Equity Act of 2021 -SB 52/HB78 | 2018: Maryland Health Care Commission – Mortality Rates of African American Infants and Infants in Rural Areas – Study - HB 716/SB 266 |  |
| 2021: Maryland Health Equity Resource Act - SB172/HB463 | 2020: Public Health - Maternal Mortality Review Program - Stakeholders - HB286 | 2021: Maryland Behavioral Health and Public Safety Center of Excellence - Establishment - HB 1280 | 2021: Maryland Office of Minority Health and Health Disparities and Maryland Health Care Commission – Reporting Requirements - SB796 | 2018: Task Force on the Social Determinants of Health in Baltimore City - SB444 |  |
| 2021: The Shirley Nathan–Pulliam Health Equity Act of 2021 -SB 52/HB78 | 2021: Public Health – Implicit Bias Training and the Office of Minority Health and Health Disparities-SB5/HB28 | 2021: Recovery for the Economy, Livelihoods, Industries, Entrepreneurs, and Families (RELIEF) Act -SB 496 | 2021: Maryland Health Equity Resource Act - SB172/HB463 | 2020: Health Services Cost Review Commission - Community Benefits - Reporting - HB1169 |  |
| 2021: Public Health – Implicit Bias Training and the Office of Minority Health and Health Disparities-SB5/HB28 | 2021: Maryland Behavioral Health and Public Safety Center of Excellence - Establishment - HB 1280 |  | 2021: Maryland Food System Resiliency Council - HB831 | 2021: The Shirley Nathan–Pulliam Health Equity Act of 2021 -SB 52/HB78 |  |
| 2020: Public Health - Maternal Mortality and Morbidity - Implicit Bias Training and Study - HB837 | 2021: Maryland Food System Resiliency Council - HB831 |  |  |  |  |
| 2020: Public Health - Maternal Mortality Review Program - Stakeholders - HB286 |  |  |  |  |  |
